# Surgeon, staff, and patient radiation exposure in minimally invasive transforaminal lumbar interbody fusion: impact of 3D fluoroscopy-based navigation partially replacing conventional fluoroscopy: study protocol for a randomized controlled trial

**DOI:** 10.1186/s13063-015-0690-5

**Published:** 2015-04-09

**Authors:** Ulrich Hubbe, Ronen Sircar, Christian Scheiwe, Christoph Scholz, Evangelos Kogias, Marie Therese Krüger, Florian Volz, Jan-Helge Klingler

**Affiliations:** Department of Neurosurgery, Freiburg University Medical Center, Breisacher Str. 64, D-79106 Freiburg, Germany

**Keywords:** Radiation exposure, Intraoperative imaging, Minimally invasive surgery, Navigation, Lumbar fusion, Spine

## Abstract

**Background:**

Some symptomatic degenerative conditions of the lumbar spine may be treated with spinal fusion if conservative treatment has failed. The minimally invasive technique of transforaminal lumbar interbody fusion (MIS TLIF) is increasingly used but has been found to generate increased radiation exposure to the patient and staff. Modern three-dimensional (3D) C-arm devices are capable of providing conventional two-dimensional fluoroscopic images (x-rays) as well as 3D image sets for intraoperative navigation. This study was designed to compare the radiation exposure between these two intraoperative imaging techniques in MIS TLIF procedures.

**Methods:**

This study is a randomized controlled trial. Forty participants scheduled to undergo monosegmental MIS TLIF will be recruited and randomly allocated to one of two groups with respect to the applied intraoperative imaging technique: conventional fluoroscopy (FLUORO group) and 3D fluoroscopy-based navigation combined with conventional fluoroscopy (NAV group). Furthermore, patients scheduled to undergo bisegmental MIS TLIF during the recruitment period for monosegmental MIS TLIF will be assessed for eligibility and will be randomly assigned separately. The primary endpoint is the radiation exposure to the surgeon and is measured by dosimeter readings. Secondary endpoints are the radiation exposure to the assistant surgeon, scrub nurse, anesthetist, patient, and C-arm as well as radiation exposure in relation to the body mass index of the patient.

**Discussion:**

Results of this randomized study will help to compare the radiation exposure to the operating staff and patient during MIS TLIF procedures using conventional fluoroscopy versus 3D fluoroscopy-based navigation combined with conventional fluoroscopy. Furthermore, recommendations regarding the appropriate use of the investigated intraoperative imaging techniques will be made to improve radiation protection and to reduce radiation exposure.

**Trial registration:**

Registration number of the German Clinical Trials Register: DRKS00004514. Registration date: 11 August 2012.

## Background

Lumbar spinal fusion is used to treat a variety of symptomatic degenerative deformities and instabilities [1]. The traditional open surgical technique includes a long skin incision with dissection and retraction of paravertebral muscles, thereby exposing the bony structures of the posterior spine. In 2003, Foley *et al.* [2] first described a minimally invasive technique for transforaminal lumbar interbody fusion (MIS TLIF) using tubular retractors under fluoroscopic guidance. A recent meta-analysis showed that MIS TLIF resulted in less blood loss and shorter hospital stay but increased x-ray exposure time compared with the open TLIF technique [3]. Modern three-dimensional (3D) C-arm devices are capable of providing conventional two-dimensional (2D) fluoroscopic images (x-rays) as well as 3D image sets for intraoperative navigation [4,5]. The main principle of intraoperative C-arm-based 3D imaging is an automated orbital rotation of the C-arm around the patient who has already been draped and positioned on the operating table. During this rotational scan, a 3D image data set is obtained from multiple successive 2D fluoroscopic images [4,5]. The 3D scan is performed after the staff has left the operating room. Additionally using navigated instruments and a navigation device, the surgeon can then perform accurate screw placement without additional radiation exposure.

The radiation exposures of MIS TLIF procedures using conventional 2D fluoroscopic images and 3D fluoroscopy-based navigation have not been systematically compared so far. Thus, the purpose of this study is to compare the radiation exposure to the surgeon, assistant surgeon, scrub nurse, anesthetist, and patient in monosegmental and bisegmental MIS TLIF using conventional fluoroscopy (FLUORO group) and 3D fluoroscopy-based navigation (NAV group) as intraoperative imaging techniques.

## Methods

### Design

This study is a randomized controlled trial to assess the intraoperative radiation exposure during monosegmental and bisegmental MIS TLIF procedures to the surgeon, assistant surgeon, scrub nurse, anesthetist, and patient. The patients are preoperatively randomly assigned to one of two intraoperative imaging groups that will be used for pedicle screw placement: conventional fluoroscopy (FLUORO group) and 3D fluoroscopy-based navigation (NAV group).

### Study population

This study will focus on patients who are at least 18 years old and who have an indication for a monosegmental or bisegmental MIS TLIF procedure within L2 and S1 because of degenerative disc disease (as opposed to infectious, neoplastic, or traumatic causes).

### Inclusion and exclusion criteria

Entry inclusion and exclusion criteria (Table 1) were developed with the goal of maximizing homogeneity of participants and therefore minimizing potential biases. Adult patients with chronic low back pain of at least 3 out of 10 at rest and at least 5 out of 10 under physical strain on the visual analogue scale (VAS) are to be enrolled if conservative treatment, including physiotherapy and adequate pain medication therapy, of at least 3 months failed to improve symptoms according to the multidisciplinary treatment path for chronic back pain of our university hospital. The cause of their symptoms has to be attributable to monosegmental or bisegmental degenerative disc disease or instability, including spondylolisthesis according to Meyerding grade I and II within L2 and S1. Radiographic signs indicative for symptomatic segments concerning degenerative disc disease or instability include spondylolysis, spondylolisthesis, Modic changes, facet joint degeneration, facet joint effusion, spinal stenosis, and severe disc collapse [6].

**Table 1:** **Inclusion and exclusion criteria of the study**

**Inclusion criteria**	**Exclusion criteria**
Age of at least 18 years	Previous surgery in the index or adjacent level
Chronic low back pain (visual analogue scale at least 3 out of 10 at rest and at least 5 out of 10 under physical strain) after having failed conservative treatment for at least 3 months	Indication for fusion of more than 2 levels
Indication for monosegmental or bisegmental minimally invasive transforaminal lumbar interbody fusion due to degenerative disc disease or instability, including spondylolisthesis according to Meyerding grade I and II within L2 and S1	Spondylodiscitis, traumatic instability, osteoporotic vertebral body fractures, neoplasm, or spondylolisthesis according to Meyerding grade III and IV of the index level(s)
	Spinal scoliosis with a Cobb angle of more than 10° in the index level(s)

Previous surgery in the index or adjacent level is an exclusion criterion since performing MIS TLIF might be more demanding because of scarring and changes of the individual anatomy. This might significantly increase x-ray exposure and therefore bias radiation exposure between the treatment groups. Likewise, patients showing a spinal scoliosis with a Cobb angle of more than 10° in the index level(s) are to be excluded. Moreover, patients with spondylodiscitis, traumatic instability, osteoporotic vertebral body fractures, neoplasm, or spondylolisthesis according to Meyerding grade III and IV are to be excluded.

### Recruitment procedures

**Figure 1:**
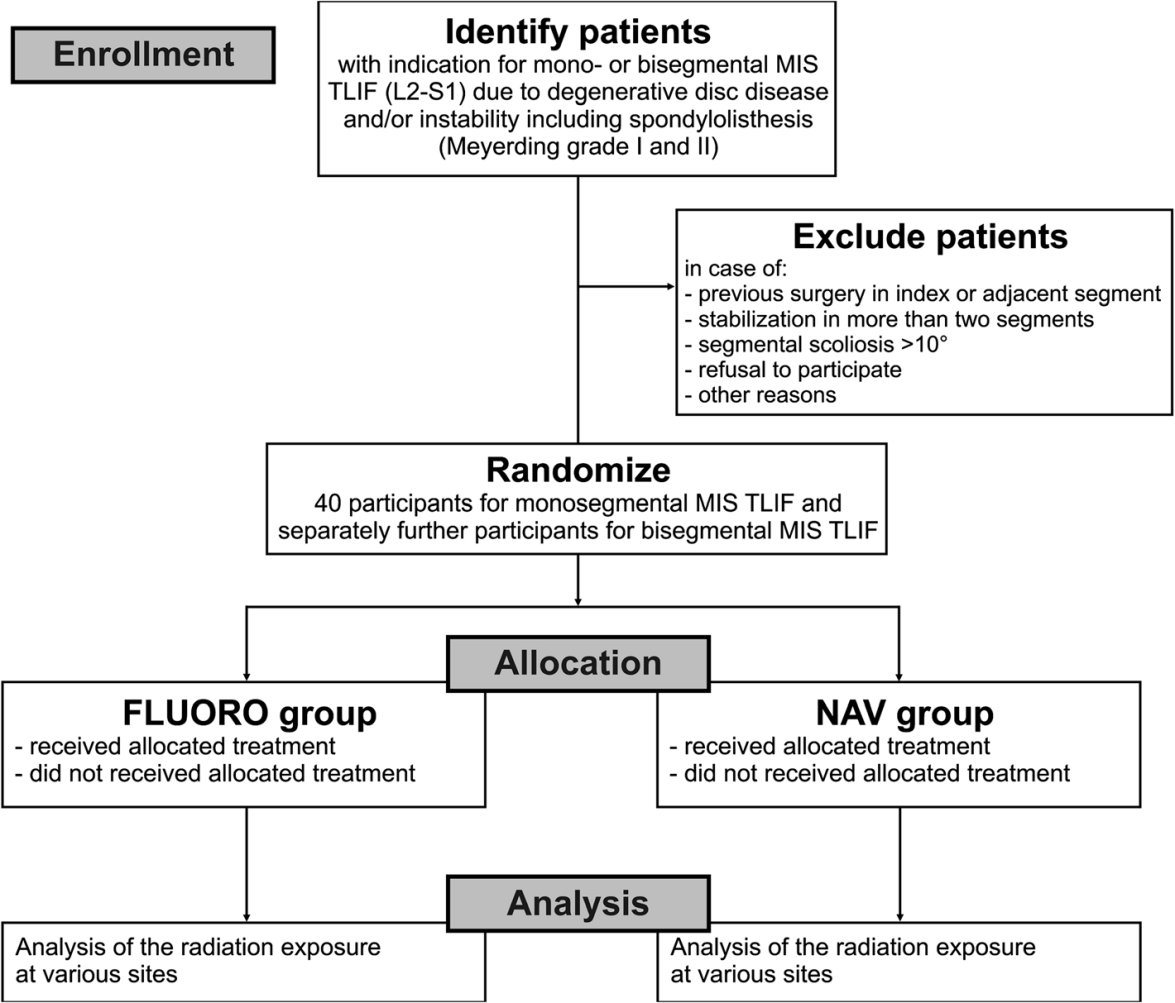
**Study design.** The figure shows the process of recruitment, randomization to treatment, and analysis of radiation exposure. FLUORO group, conventional fluoroscopy group; MIS TLIF, minimally invasive transforaminal lumbar interbody fusion; NAV group, three-dimensional fluoroscopy-based navigation group.

The Department of Neurosurgery of the University Hospital in Freiburg has been performing MIS TLIF procedures routinely for years by using conventional fluoroscopy and 3D fluoroscopy-based navigation. Participants for this study will be recruited primarily from the neurosurgical outpatient clinic. Their eligibility will be proven according to the inclusion/exclusion criteria (Table 1). If eligible, participants are informed about the study protocol. Once written consent is obtained, participants are randomly assigned to one of the two treatment groups (Figure 1).

### Randomization to treatment groups

Participants will be randomly assigned by using ‘RITA - Randomization In Treatment Arms’ computer software (StatSol, Sereetz, Germany). Participants were randomly assigned in permuted blocks of four and six, according to computer-generated random numbers, to undergo MIS TLIF using either conventional fluoroscopy (FLUORO group) or 3D fluoroscopy-based navigation (NAV group). Separate randomization files are created for monosegmental and bisegmental MIS TLIF. The randomization software ensures that the treatment allocation cannot be viewed prior to randomization and cannot be changed after randomization.

### Study treatments

#### Dosimeter setup

**Figure 2:**
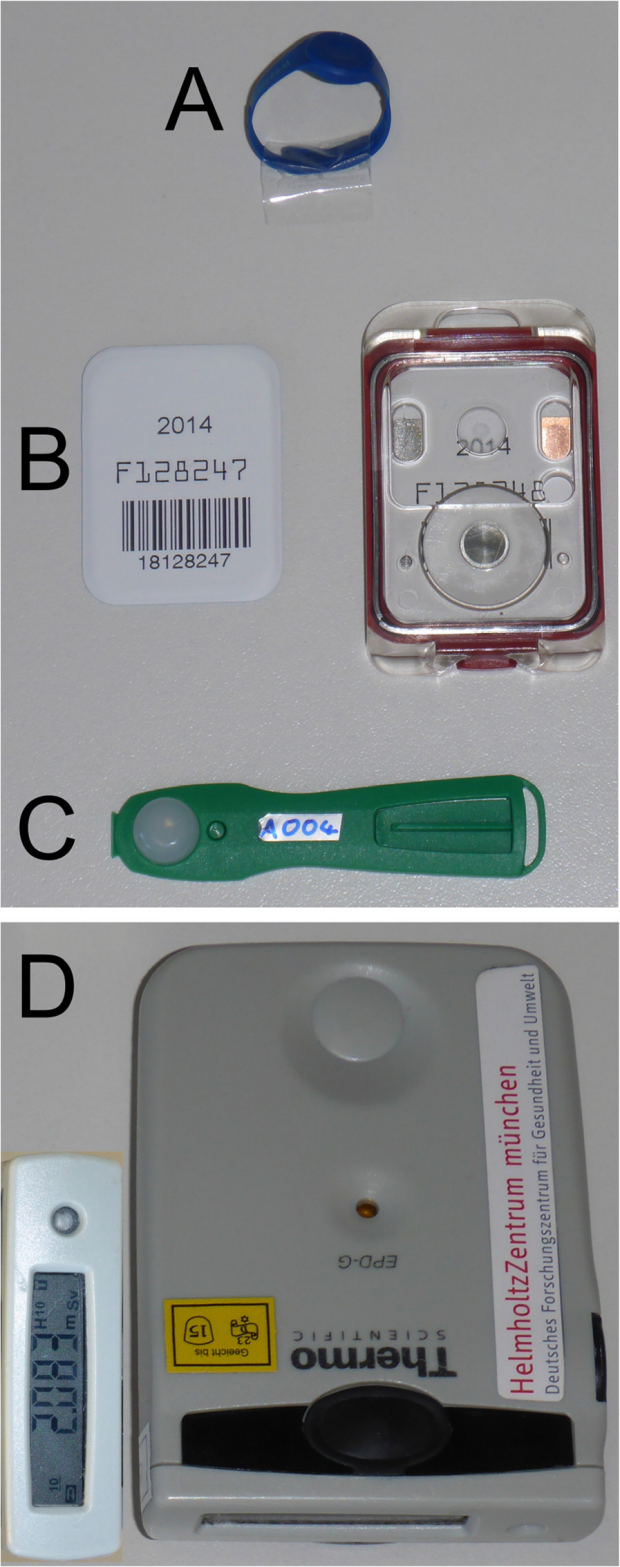
**Overview of dosimeter types used. (A)** Ring dosimeter. **(B)** Film dosimeter with cassette. **(C)** Eye lens thermoluminescence dosimeter. **(D)** Electronic personal dosimeter with superimposed digital display.

Dosimeters are attached to the surgeon, assistant surgeon, scrub nurse, anesthetist, patient, and C-arm in the operating room (Table 2). Four different types of dosimeters are used (Figure 2). Film dosimeters (AWST-FILM GD 60, *H*_p_(10); Helmholtz Zentrum München, part of the German Research Center for Environmental Health, Personal Monitoring Service, Munich, Germany) are attached to the trunk of the surgeon, assistant surgeon, scrub nurse, anesthetist, patient, and C-arm. Eye lens thermoluminescence dosimeters (EYE-D, *H*_p_(3); Radcard, Krakow, Poland) are attached to the surgeon, assistant surgeon, and scrub nurse. Ring dosimeters (AWST-TL-TD 60, type W; Helmholtz Zentrum München) are tightened around the ring fingers of the surgeon. Moreover, an Electronic Personal Dosimeter (EPD Mk2, *H*_p_(10) mode; Thermo Scientific, Schwerte, Germany) is installed at the flat panel detector of the C-arm to read radiation exposures at predetermined time points during surgery. This dosimeter location approximately corresponds to a surgeon who does not step back from the radiation source when applying x-rays, and this is regularly the case in most operating rooms. With the EPD, we have radiation exposure readings at any time of the operation and thus have the possibility to approximately relate radiation exposures to certain steps of the operation.

**Table 2:** **Survey of the intraoperative dosimeter setup**

	**Location**	**Dosimeter type**	**Dosimeter lead protected?**
Surgeon	Left ring finger	Ring dosimeter	Unprotected
	Right ring finger	Ring dosimeter	Unprotected
	Middle forehead	Eye lens dosimeter	Unprotected
	Under lead goggles (left glass)	Eye lens dosimeter	Protected
	Thyroid gland, above lead shield	Film dosimeter	Unprotected
	Thyroid gland, under lead shield	Film dosimeter	Protected
	Left chest, above lead apron	Film dosimeter	Unprotected
	Left chest, under lead apron	Film dosimeter	Protected
	Gonad, under lead apron	Film dosimeter	Protected
	Right knee, under lead apron	Film dosimeter	Protected
	Back, between shoulder blades, above lead apron	Film dosimeter	Unprotected
Assistant surgeon	Middle forehead	Eye lens dosimeter	Unprotected
	Left chest, above lead apron	Film dosimeter	Unprotected
	Left chest, under lead apron	Film dosimeter	Protected
Scrub nurse	Middle forehead	Eye lens dosimeter	Unprotected
	Left chest, above lead apron	Film dosimeter	Unprotected
	Left chest, under lead apron	Film dosimeter	Protected
Anesthetist	Left chest, above lead apron	Film dosimeter	Unprotected
	Left chest, under lead apron	Film dosimeter	Protected
Patient	Thyroid gland	Film dosimeter	Unprotected
	Chest	Film dosimeter	Unprotected
	Gonad	Film dosimeter	Unprotected
C-arm	Next to generator	EPD	Unprotected
	Next to generator	Film dosimeter^a^	Unprotected
	Next to flat panel detector	Film dosimeter^a^	Unprotected

^a^Film dosimeters on the C-arm are removed during the three-dimensional (3D) scan (NAV group, three-dimensional fluoroscopy-based navigation group) since the staff is outside the operating room during the 3D scan. EPD, electronic personal dosimeter.

Two dosimeters of each type (film dosimeter, eye lens thermoluminescence dosimeter, and ring dosimeter) are placed outside the operating room and serve as reference dosimeters. All dosimeters are provided and will be evaluated by Helmholtz Zentrum München.

#### Lead protection

The surgeon, assistant surgeon, and scrub nurse wear at least a two-piece lead apron consisting of a vest (lead equivalent values of 0.70 mm Pb in the front part and 0.25 mm Pb in the back part) and a skirt reaching below the knees (lead equivalent values of 0.50 mm Pb in the front part and 0.25 mm Pb in the back part). The surgeon additionally wears a thyroid shield (lead equivalent value of 0.50 mm Pb) and lead protective goggles (lead equivalent value of 0.75 mm Pb). The anesthetist wears a one-piece lead apron (lead equivalent value of 0.35 mm Pb in the front part).

#### Surgical technique

All MIS TLIFs are performed primarily by the first author (UH) (and by RS by proxy). The patient is positioned prone on a radiolucent table, allowing fluoroscopic images in anterior-posterior and lateral projection as well as 3D scans. Prior to draping of the patient, the projections of the pedicles were identified and marked on the skin by using fluoroscopic images. The mobile 3D C-arm device, Ziehm Vision FD Vario 3D with flat panel detector (Ziehm Imaging, Nuremberg, Germany), is used for all MIS TLIFs in this study and is set on automatic exposure control with corresponding kilovolt and milliampere values of varying magnitudes; this is usual in daily practice [7]. No continuous fluoroscopy is used. Only single fluoroscopic images are acquired (C-arm in pulsed mode with 12.5 images per second).

For insertion of transpedicular Kirschner wires in the NAV group, a navigation tracker is mounted on the spinous process of one of the index vertebras via a median skin incision of approximately 2.5 cm. Subsequently, an automated 3D navigation scan is performed in apnea by using the 3D capable C-arm Ziehm Vision FD Vario 3D. During the 3D scan, the entire staff leaves the operating room and thus has a minimum distance of 6 m from the x-ray generator and patient. The 3D image set is automatically transferred to a navigation device (for example, Cart II system device; Stryker, Freiburg, Germany: SpineMap 3D navigation), and navigation tools are referenced. Via bilateral short skin incisions (2 to 3 cm), a navigated Jamshidi needle is introduced into the target vertebras through the pedicles, and Kirschner wires are inserted through the Jamshidi needle without additional use of fluoroscopic images.

**Figure 3:**
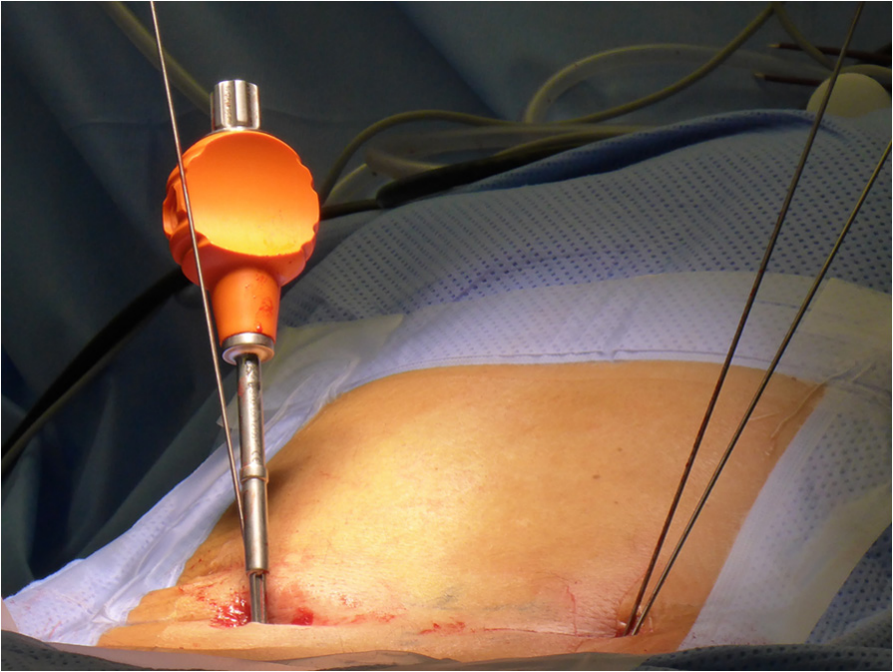
**Intraoperative view on the Kirschner wires and Jamshidi needle (orange) introduced during a minimally invasive transforaminal lumbar interbody fusion (MIS TLIF) procedure.**

In the FLUORO group, a Jamshidi needle is introduced via bilateral short skin incisions (2 to 3 cm) to locate the ideal pedicle entry point between the transverse process and lateral facet joint. This is accomplished by using tactile feedback and conventional fluoroscopic image guidance in lateral projection without exposing the anatomical structures. Then the Jamshidi needle is introduced into the target vertebras through the pedicles verified by fluoroscopic images in lateral projection when necessary, and Kirschner wires are inserted through the Jamshidi needle (Figure 3).

Since the surgeon inserts all Kirschner wires himself, he is changing sides of the table and therefore is exposed to the corresponding scatter radiation at the ipsilateral or contralateral side of the C-arm generator, respectively. Thereafter, neurophysiological monitoring is performed for safe pedicle screw placement in both treatment groups. Integrity of the pedicle wall is evaluated with monopolar and bipolar stimulation by using a tip stimulation probe with recording of the compound muscle action potentials. With monopolar stimulation, a constant current below 7 milliampere is regarded as suspicious of pedicle perforation [8], and revising the trajectory has to be considered. Then the Kirschner wires are drawn back under fluoroscopic image guidance in lateral projection, so that their tips are positioned at the posterior border of the vertebral bodies. Their intrapedicular position is controlled by using fluoroscopic image guidance in anterior-posterior projection.

The next step is to insert and mount the non-expandable tubular retractor system METRx (Medtronic, Minneapolis, MN, USA) on the facet joint for the MIS TLIF approach. In the NAV group, the facet joint is located by using the navigated Jamshidi needle without additional use of fluoroscopic images. In the FLUORO group, the localization with the Jamshidi needle has to be performed by using fluoroscopic image guidance in lateral projection. Thereafter, in both treatment groups, the transmuscular approach is created by using METRx dilators. Finally, a METRx operation tube (diameter of 20 mm) is inserted and fixed to the table. During the following transforaminal access including facetectomy, unilateral or bilateral decompression of the spinal canal via unilateral partial hemilaminectomy, nucleotomy and implantation of the TLIF cage (with additional insertion of autologous bone into the intervertebral disc space, which has been collected during facetectomy), the surgeon uses the operating microscope from the ipsilateral side. Implantation of the cage is performed under fluoroscopic image guidance in lateral projection.

Subsequent screw insertion via the Kirschner wires and rod insertion (using CD Horizon Sextant II, CD Horizon Sextant Solera, or CD Horizon Longitude; Medtronic) is performed identically in both treatment groups without routine use of conventional fluoroscopy. If bone density is considered to be low, cement augmentation of the vertebras is performed via perforated screws under fluoroscopic image control in lateral projection [9]. Final control is made by using fluoroscopic image guidance in lateral and anterior-posterior projection.

The following guidelines must be followed as radiation protection principles: (a) continuous fluoroscopy is not used, (b) the distance between the patient’s surface and the flat panel detector is minimized, (c) beam collimation is used whenever possible, (d) a distance from the radiation source and patient’s surface (as the origin of the Compton scatter) is maintained whenever possible, (e) the hand is removed from the path of the x-ray beam when holding an instrument, and (f) no staff member stays in the operating room during acquisition of the 3D scan.

### Data collection

In this study, the primary endpoint is the radiation exposure to the surgeon during monosegmental MIS TLIFs in the FLUORO and NAV groups. The secondary endpoints are the radiation exposures to the assistant surgeon, scrub nurse, anesthetist, patient, and C-arm as well as radiation exposure in relation to the body mass index of the patient in the FLUORO and NAV groups.

### Primary endpoint measurement

Radiation exposures are determined by the applied dosimeters. Dosimeter readings are corrected with the readings of the reference dosimeters, which are positioned outside the operating room.

### Secondary endpoint measurement

Radiation exposures are determined as described above. Furthermore, radiation exposure of the EPD at the flat panel of the C-arm is read on the display at defined time points during surgery. Body mass index is obtained preoperatively and is defined as the patient’s weight in kilograms divided by the square of the height in meters.

### Ethics statement

The study protocol has been approved by the institutional ethics committee of the University of Freiburg (Germany), where the monocentric study takes place (reference number 431/12). The trial is registered with the German Clinical Trials Register (DRKS00004514) [10]. Written consent will be obtained from each participant before randomization and treatment.

### Statistical issues

#### Sample size

The estimation of the sample size is based on data provided in the literature. We determined a recruiting period of 3 years for inclusion of 40 participants with monosegmental MIS TLIF procedures.

For estimating the radiation exposure in the FLUORO group, we made the following assumptions: Bindal *et al.* [11] stated an average radiation exposure of 266 μSv to the surgeon (at the lead protected waist) during an MIS TLIF procedure using conventional fluoroscopy with an average radiation time of 101.4 seconds. A retrospective survey in our department showed an average radiation time of 55.75 seconds in 16 consecutive MIS TLIF procedures using conventional fluoroscopy. Comparing this radiation time with the data of Bindal *et al.* [11], we assume a calculated radiation exposure to the surgeon of 146.2 μSv (266 μSv × 55.75 seconds/101.4 seconds) in our department.

For estimating the radiation exposure in the NAV group, we made the following assumptions: We estimate that 12 conventional fluoroscopic images are obtained for one monosegmental MIS TLIF procedure in attendance of the surgeon (the 3D fluoroscopy-based image data set is attained in an automated fashion after the staff has left the operating room, thus not causing radiation exposure to the staff). Rampersaud *et al.* [12] stated an average radiation time of 1.1 seconds per fluoroscopic image. With this data, we assume a radiation exposure to the surgeon of 34.6 μSv (12 images × 1.1 seconds/image × 266 μSv/101.4 seconds) during a monosegmental MIS TLIF procedure using 3D fluoroscopy-based navigation.

Based on the above-mentioned assumptions, we used radiation exposures of 150 μSv (FLUORO group) and 50 μSv (NAV group) for calculation of the estimated sample size. The alpha level was set to 0.05, and the level of statistical power for calculating the sample size was set to 0.8. This power analysis led to a sample size of n = 17 for each group for monosegmental MIS TLIF procedures applying the two-tailed *t* test for independent samples. We aim to include 20 patients with monosegmental MIS TLIF procedures in each group.

#### Statistical analysis

The primary endpoint (radiation exposure to the surgeon) will be tested at the 0.05 level. Results will be expressed as means with standard deviations. Analysis of independent continuous quantitative variables between groups will be performed by using the two-tailed Student’s *t* test. Pearson’s correlation test will be used to determine correlations between two variables. Statistical comparisons for categorical values between groups will be performed by using the two-tailed Fisher exact test. Prism 6 for Mac (GraphPad Software Inc., La Jolla, CA, USA) will be used as statistical software.

## Discussion

Since there is no threshold dose according to the stochastic model for radiation injuries, spine surgeons should apply x-rays thoughtfully and sparingly [13,14]. Minimally invasive fusion techniques, in particular, can result in increased x-ray exposure times [3]. Bearing in mind that surgeon and staff are exposed to radiation during their daily routine, one has to find solutions to reduce the occupational radiation exposure. Radiation protection principles like wearing adequate lead equipment (including thyroid shield and lead glass goggles), application of beam collimation, and keeping a distance from the radiation source should be self-evident [7]. Further reduction might be obtained by using 3D fluoroscopy-based navigation since the staff is leaving the operating room for acquisition of the 3D image scan, thus avoiding additional radiation exposure. In contrast, application of 3D fluoroscopy-based navigation might increase radiation exposure to the patient. This randomized study aims to compare the radiation exposure to the operating staff and patient during MIS TLIF procedures using conventional fluoroscopy or 3D fluoroscopy-based navigation.

## Trial status

This trial is currently recruiting participants. Enrollment is expected to be finished in 2016.

## Abbreviations

2D: Two-dimensional
3D: Three-dimensional
EPD: Electronic personal dosimeter
FLUORO group: Conventional fluoroscopy group
MIS TLIF: Minimally invasive transforaminal lumbar interbody fusion
NAV group: Three-dimensional fluoroscopy-based navigation group
Pb: Lead
TLIF: Transforaminal lumbar interbody fusion
